# Identification of 12 genetic loci associated with human healthspan

**DOI:** 10.1038/s42003-019-0290-0

**Published:** 2019-01-30

**Authors:** Aleksandr Zenin, Yakov Tsepilov, Sodbo Sharapov, Evgeny Getmantsev, L. I. Menshikov, Peter O. Fedichev, Yurii Aulchenko

**Affiliations:** 1 Gero LLC, Novokuznetskaya street 24/2, Moscow, Russia 119017; 2 Novosibirsk State University, Pirogova 2, Novosibirsk, Russia 630090; 3 Institute of Cytology and Genetics SB RAS, Lavrentyeva ave. 10, Novosibirsk, Russia 630090; 4 National Research Center “Kurchatov Institute”, 1, Akademika Kurchatova pl., Moscow, Russia 123182; 5 Moscow Institute of Physics and Technology, Institutskii per. 9, Dolgoprudny, Moscow Russia 141700; 6 PolyOmica, Het Vlaggeschip 61, 5237PA ‘s-Hertogenbosch, The Netherlands; 7 Centre for Global Health Research, Usher Institute of Population Health Sciences and Informatics, University of Edinburgh, Teviot Place, Edinburgh, Scotland EH8 9AG UK

## Abstract

Aging populations face diminishing quality of life due to increased disease and morbidity. These challenges call for longevity research to focus on understanding the pathways controlling healthspan. We use the data from the UK Biobank (UKB) cohort and observe that the risks of major chronic diseases increased exponentially and double every eight years, i.e., at a rate compatible with the Gompertz mortality law. Assuming that aging drives the acceleration in morbidity rates, we build a risk model to predict the age at the end of healthspan depending on age, gender, and genetic background. Using the sub-population of 300,447 British individuals as a discovery cohort, we identify 12 loci associated with healthspan at the whole-genome significance level. We find strong genetic correlations between healthspan and all-cause mortality, life-history, and lifestyle traits. We thereby conclude that the healthspan offers a promising new way to interrogate the genetics of human longevity.

## Abstract (web summary)

Aleksandr Zenin et al. present a genome-wide association study and genetic risk model for human healthspan, the length of morbidity-free life. They identify 12 loci associated with healthspan and osbserve genetic correlations between healthspan and life-history and lifestyle traits, such as obesity and smoking.

## Introduction

Age is the most important single risk factor for multiple diseases, see, e.g., ref. ^1^. Likewise, extreme longevity in human cohorts is associated with a delayed incidence of diseases: Kaplan-Meyer curves of disease-free survival, stratified by age, demonstrate a consistent delay in the onset of age-related diseases with increasing age of survival^2^. Therefore, the emerging premise is that aging itself is the common driver of chronic diseases and conditions that limit the functional and disease-free survival^3^. Healthy and morbidity-free lifespan, often termed “healthspan”, is thus a promising phenotype for longevity research^4^ and possibly a target for future anti-aging interventions^3,5^. The thorough delineation between the healthspan and lifespan is more than of academic interest: the last century saw a dramatic increase in lifespan, not necessarily followed by a matching improvement in the healthspan^6^.

Genomics provide a hypothesis-free approach to study the biology of complex traits, including aging^5^. The increasing number of available genomes of very old people^7–9^, though representing a rather specific and a relatively small sub-group of exceptionally successfully aging individuals, can provide an insight into the genetic architecture of exceptional life-spans and health-spans by use of Genome-Wide Association Studies (GWAS). While such studies suggested a fair number of loci, the *APOE* locus is probably among the few consistently implicated in multiple studies, see ref. ^10^ for a review. GWAS of the disease-free survival has been performed in relatively large cohorts (*n* = 25,007), however, without producing genome-wide significant associations^11^, highlighting the complexity of healthspan phenotype. Further gains can be naturally achieved by increasing the population size with the help of proxy phenotypes, such as a search for genetic variants that predispose one to age-related disease and hence are depleted in long-lived persons compared to controls^8^. Another promising alternative involves GWAS of parental lifespans^12–14^.

In this paper, we focused on aging and morbidity in mid-life using clinical histories for over 300,000 people, aged 37 to 73, and participating in the UK Biobank (UKB) cohort. We checked the for incidence of chronic diseases and identified a cluster of the top eight morbidities strongly associated with age after the age of 40 and ranked by the number of occurrences. We observed that the risk of the selected diseases increases exponentially at similar rates. The corresponding doubling time is approximately eight years, close to the mortality risk doubling time from Gompertz law of mortality^15^. The close association between disease and mortality risk dynamics suggests the possibility of a single underlying mechanism, that is aging. We hypothesize that the incidence of the selected diseases is therefore a natural measure of the organism resilience and hence of aging process progression. Accordingly, the disease-free survival, the healthspan, is expected to be a useful phenotype, directly associated with the rate of aging. To reveal the genetic determinants of the healthspan, we built a proportional hazards model to predict the age corresponding to the incidence of the first disease from the “Gompertzian cluster” depending on an individual’s age, gender, genetic variation, and a number of more “technical” covariates. We used the sub-population of 300,447 genetically confirmed white British ancestry individuals (hereafter referred to as GCW-British) as a discovery cohort for a GWAS and identified 12 loci associated with healthspan at the whole-genome level of significance. The genetic signature of healthspan has high and significant genetic correlations with GWAS of obesity, type 2 diabetes, coronary heart disease, traits related to metabolic syndrome, and all-cause mortality (as derived from parental survival). We conclude by noting that the healthspan phenotype offers a promising new way to investigate human aging by exploiting the data from large cohorts of living individuals with rich clinical information.

## Results

### Healthspan in UK Biobank

**Fig. 1:**
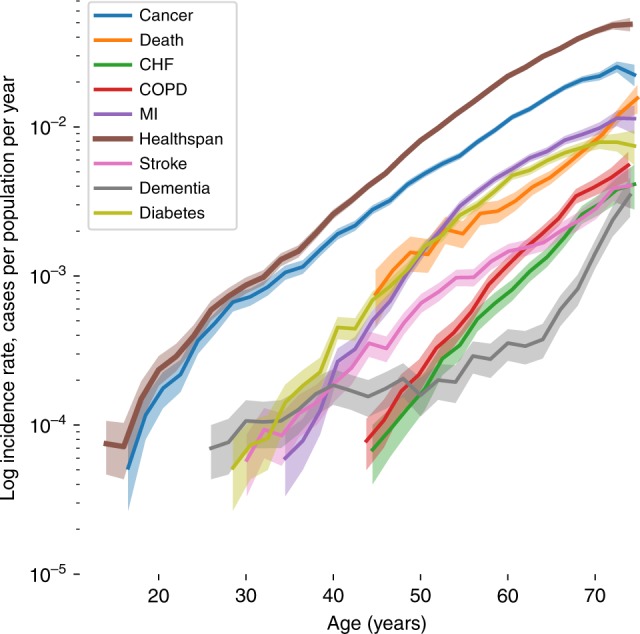
The incidence of the most prevalent chronic diseases, risk of death (the mortality rate) and healthspan for UKB participants. The disease incidence increases approximately exponentially with age at approximately the same rates. Disease incidence rates are calculated independently, participants that have more than one condition during follow-up period are counted for every disease they have, except for healthspan which is defined as the first event occurred. Shaded area represents 95% confidence interval

We studied the dynamics of disease incidence using the clinical data available from the UKB. We followed^2^ and selected the top eight morbidities strongly associated with age after the age of 40, having a discrete clinically apparent outcome (for example, hypertension was not included because if present, it was probably being treated with medication, thus markedly decreasing its effect upon morbidity) and ranked by the number of occurrences. The shortlist included Congestive Heart Failure (CHF), Myocardial Infarction (MI), Chronic Obstructive Pulmonary Disease (COPD), stroke, dementia, diabetes, cancer, and death (Table 1, Supplementary Data 1). The risks of the selected conditions were found to increase exponentially with age at approximately the same rates (Fig. 1; see Supplementary Data 2 and Methods section Incidence of diseases calculation from UKB data for details). The characteristic doubling time is approximately seven to eight years. The risk of death in the dataset also grows exponentially with age following empirical Gompertz mortality law^15,16^. The manifested similarity between the diseases and the mortality risk doubling time suggest that the most plausible single unifying mechanism behind the risk acceleration with age is aging itself.

**Table 1:** Number of events derived from clinical and interview data for selected diseases and combined data (see Methods section for details) used for healthspan calculation for total 300,447 participants

	Clinical data		Interview data		Combined data	
	Events	%	Events	%	Events	%
Cancer	66,214	51.4	41,485	48.6	74,172	51.3
Diabetes	20,019	15.5	23,134	27.1	26,026	18.0
MI	25,649	19.9	10,150	11.9	24,751	17.1
Stroke	4731	3.7	6070	7.1	6902	4.8
COPD	6211	4.8	1484	1.7	5881	4.1
Dementia	769	0.6	2889	3.4	2706	1.9
Death	2411	1.9	0	0.0	2399	1.7
CHF	2850	2.2	231	0.3	1883	1.3

We chose to define healthspan as the age of the onset of the first disease from our list of the selected “Gompertzian” diseases or death. As expected, the first morbidity incidence rate also increases exponentially with age (see the brown “healthspan” line in Fig. 1), the corresponding doubling time matches the mortality, and the specific disease risk doubling times. In the UKB cohort, healthspan is ended by cancer in more than half of the cases, followed by diabetes and MI, and very rarely by death, see Table 1. These three diseases alone account for over 86% of the end of healthspan period (although cancer can be considered a large variety of diseases). Death occurs later in life and follows the end of the disease-free survival by approximately a decade (there are less than 2% cases when death precedes incidence of any of the chronic diseases). The total number of the participants with one or more chronic diseases, 84,949, is dramatically larger than that of death events, 8365, out of 300,447 study population (see below for the GWAS inclusion criteria). Pearson correlation between healthspan and lifespan event time in 8365 participants for whom both events were available was *r* = 0.726 (at the number of deaths preceding the chronic diseases in the dataset, the inclusion of death in the definition of healthspan does not substantially contribute to the correlation estimate). Iterative multiple imputation method^17^ that is often used for comparison of survival data gives *ρ* = 0.573 (0.530–0.613 95%CI).

### Genome-wide association study design

Next, we assumed there is a group of genetic factors, predisposing individuals to the early onset of chronic diseases and identified gene-variants associated with the shorter healthspan. Since the incidence of the first morbidity risk grows exponentially with age, we propose to employ the Cox-Gompertz proportional hazard model (see, e.g., ref. ^18^) to test statistical associations between specific genes and disease risks. In subsection Cox-Gompertz proportional hazards model and healthspan we explain how to use a maximum likelihood version of Cox-Gompertz model to predict the age corresponding to the end of healthspan for each study participant.

We started by characterizing each of the 300,447 individuals in the study cohort by sex and age, followed by the technical (genotyping batch, assessment center), and the ethnicity-related genetic variables (40 first genetic principal components). A maximum likelihood optimization produced the best fit proportional hazards model parameters. The morbidity incidence growth rate was found to be 0.098 per year, which corresponds to a doubling time of seven years and is compatible with the mortality rate doubling time of approximately eight years from Gompertz mortality law. As expected, being male is a significant risk factor (log-hazard ratio, log(HR) = 0.26 at the significance level of *p* = 5 × 10^−301^), with a corresponding healthspan difference of approximately three years. The genetic principal components PC4 and PC5, and some of the assessment center labels were also highly significantly associated with the healthspan (see Supplementary Data 3 and Methods, Cox-Gompertz proportional hazards model and healthspan, for details). From these numbers, we observed that human mortality and the first morbidity incidence follow a version of Gompertz law. The average healthspan can be readily estimated from the Gompertz model parameters as 72 years, which is 14 years less than the Cox-Gompertz lifespan estimate for the same cohort. Since we did not expect a substantial effect on healthspan from any of the individual gene-variants, the effect sizes and the significance testing could be performed using a form of linear regression to the Martingale residual of the Cox-Gompertz model above, see subsection Gene variant-healthspan association testing. In this study, we limited the discovery association screen to the study cohort (300,447 individuals) with available genetic information with 11,309,218 imputed autosomal variants.

### GWAS results

**Fig. 2:**
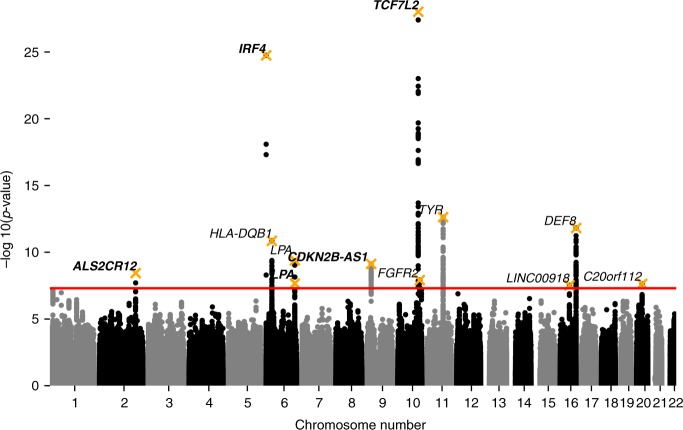
Discovery GWAS of healthspan in GCW-British individuals. The trait is a form of Martingale residual of the Cox-Gompertz proportional hazards model of healthspan as described in section Cox-Gompertz proportional hazards model and healthspan. The loci are tagged by SNPs from Table 2, labeled by the nearest gene symbol, replicated SNPs marked in bold

A total of 394 SNPs at 14 loci achieved a genome-wide significance threshold of *p* < 5 × 10^−8^ (Supplementary Data 4). Using the median estimator, the genomic control inflation parameter *λ*^19^ was 1.18. The LD score regression^20^ yielded the healthspan heritability of 0.102 (se = 0.009), and the LD score regression intercept was 1.053 (se = 0.008, ratio = 0.24). After adjusting the results of the discovery GWAS for genomic control of 1.053, a total of 328 SNPs positioned in 12 loci remained statistically significant at the genome-wide level (Fig. 2). The conditional and joint analysis (COJO) as implemented in the program GCTA^21^ confirmed that all the regions were independent except a locus on chromosome 6, at 161 Mb (Supplementary Data 5). We detected two signals in this locus (rs140570886 and rs10455872) that had linkage disequilibrium *R* = −0.04 and *D*′ = 1.0. The distance between these SNPs was 3kbp, and they had relatively small frequencies (0.08 and 0.016, respectively).

For replication, we used a combination of the UK Biobank participants not included in the discovery set whose self reported ancestry was European (white, data-field 21000, *n* = 81,099), African (*n* = 3073), South Asian (Indian, Pakistani, and Bangladeshi; *n* = 6921), Chinese (*n* = 1422) and Caribbean (*n* = 3799). Using meta-analysis for the selected subsets (total *N* = 96,313), we performed the analysis on the 12 genome-wide significant SNPs for the replication group (Supplementary Data 5). Of the 12 SNPs, for all but one, the same allele turned out to be risk-increasing both in the discovery and in the replication samples. Five associations were significant after correction for multiple testing with *p* < (0.05/12). We subsequently refer to these five SNPs as ‘replicated’.

### Genetic correlation analysis

**Fig. 3:**
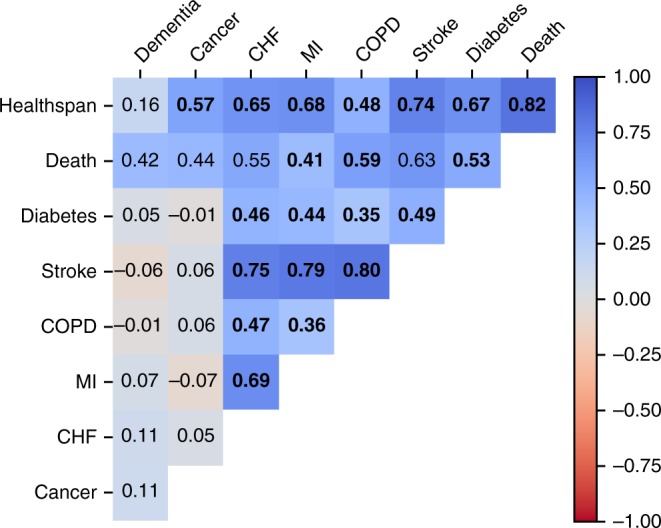
Genetic correlation between GWAS of the healthspan and the diseases used to produce the healthspan phenotype in the UKB discovery cohort. The significant correlations marked in bold (*p* < 0.05 after Bonferroni correction)

First we checked the genetic correlations between the healthspan GWAS results and the genetic signatures of the individual diseases used to build the healthspan phenotype. To do this, we produced a series of independent GWAS of the age at onset of the individual conditions, using the same Cox-Gompertz methodology (Fig. 3, Supplementary Data 6). The healthspan GWAS exhibits strong correlations with most of the disease traits, with the notable exception of dementia (see the discussion below). Interestingly, the mortality, stroke, CHF, diabetes, and MI traits showed higher genetic correlations with healthspan, than did cancer, even though cancer was the most frequent healthspan-terminating event in our study.

We checked if any of the the gene variants associated with shorter healthspan in our study could be common risk factors for multiple diseases. Since cancer had approximately the same prevalence as all the other diseases combined, we tested the SNPs from Table 2 in Cox proportional hazard models of incidence of all cancers, on one hand, and the first incidence of diabetes, MI, stroke, COPD, dementia or death, on the other hand (see Supplementary Data 7). Of 12 tested SNPs, 5 and 4 SNPs turned out to be independent cancer and non-cancer disease risk factors, whereas the other 3 (rs1049053, rs1126809, and rs159428) appeared to be significantly associated with both outcomes.

**Table 2:** Variants, tagging regions, significantly associated with the first morbidity hazard (end of healthspan) in 300,447 GCW-British individuals, and results of replication in 96,313 individuals

SNP	Chr	Position (bp)	EA	RA	EAF	beta	*P*	*β*_rep_	*P*_rep_
**rs10197246**	**2**	**202,204,741**	**C**	**T**	**0.734**	−**0.033**	**3.67e-09**	−**0.035**	**2.43e-04**
**rs12203592**	**6**	**396,321**	**T**	**C**	**0.214**	**0.063**	**1.80e-25**	**0.043**	**2.10e-05**
rs1049053	6	32,634,405	T	C	0.671	0.037	1.40e-11	0.013	1.46e-01
rs10455872	6	161,010,118	G	A	0.081	0.057	4.11e-10	0.027	1.19e-01
**rs140570886**	**6**	**161,013,013**	**C**	**T**	**0.016**	**0.116**	**2.18e-08**	**0.131**	**4.09e-04**
**rs7859727**	**9**	**22,102,165**	**T**	**C**	**0.488**	**0.031**	**7.41e-10**	**0.041**	**1.52e-06**
**rs34872471**	**10**	**114,754,071**	**C**	**T**	**0.292**	**0.061**	**9.73e-29**	**0.062**	**2.86e-11**
rs2860197	10	123,351,302	A	G	0.613	−0.029	1.22e-08	−0.007	4.47e-01
rs1126809	11	89,017,961	A	G	0.304	0.04	2.35e-13	0.017	7.59e-02
rs4784227	16	52,599,188	T	C	0.24	0.032	3.02e-08	0.018	7.75e-02
rs4268748	16	90,026,512	C	T	0.311	0.038	1.55e-12	0.004	6.24e-01
*rs159428*	*20*	*31,099,311*	*C*	*T*	*0.527*	*0.028*	*2.36e-08*	*−0.005*	*5.84e-01*

EA, effective (coded, tested) allele; RA, reference (non-coded) allele; EAF, effect allele frequency; *β*, regression coefficient estimate (units of measurement is log(hazard ratio) per allele); *p*, *p*-value after adjustment for genomic control; *β*_rep_, regression coefficient estimate in replication sample; *p*_rep_ *p*-value in replication sample. In bold: replicated loci. In italics: locus demonstrating opposite effect in replication

**Fig. 4:**
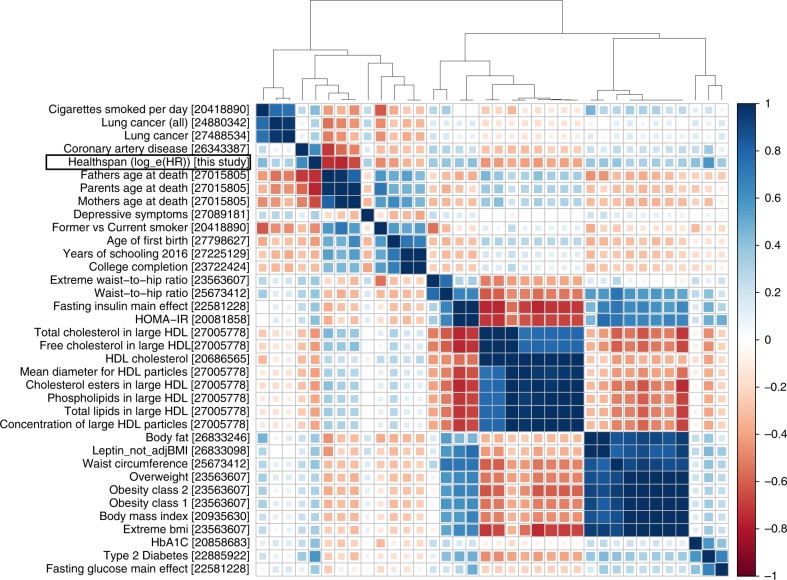
Thirty-five traits with significant and high genetic correlations with healthspan (|*r*_g_|  ≥ 0.3; *p* ≤ 4.3 × 10^−5^). PMID references are placed in square brackets. Note the absence of genetic correlation between the healthspan and Alzheimer disease traits (*r*_g_ = −0.03)

To obtain a broader insight into biological significance of our findings we analyzed genetic correlations between healthspan and 235 complex traits studied in samples other than the UK Biobank and available from the LD-hub (231 traits after removing duplicates)^22^. Overall, we observed significant genetic correlations (*p* < 0.01/231 = 4.3 × 10^−5^) between the healthspan and 46 traits (Supplementary Data 8). The strongest positive correlations (*r*_g_ > 0.4) were found in association with coronary artery disease (CAD)^23^ (*r*_g_ = 0.62), Type 2 Diabetes^24^ (*r*_g_ = 0.58), glycated hemoglobin level (HbA1C)^25^ (*r*_g_ = 0.42), cigarettes smoked per day^26^ (*r*_g_ = 0.44), and insulin resistance index (HOMA-IR)^27^ (*r*_*g*_ = 0.41). The strongest negative correlations (*r*_g_ < −0.4) were for the age of first birth^28^ (*r*_g_ = −0.43), father’s age at death, mother’s age at death, and combined parental age at death defined as a sum of standardized mother’s and father’s age at death^29^ (*r*_g_ = −0.74, −0.66, −0.76, respectively) former vs. current smoker^26^ (*r*_g_ = −0.48) and HDL related traits^30^ (cholesterol esters in large HDL, total lipids in large HDL, total cholesterol in large HDL, mean diameter for HDL particles, free cholesterol in large HDL, with *r*_g_ = −0.44, −0.41, −0.44, −0.42, and −0.43, respectively). Figure 4 summarizes the results of the clustering analysis of the top genetic correlations selected by significance and magnitude. We found, that 35 traits with large and significant genetic correlation with healthspan (|*r*_g_| > 0.3 and *p* < 4.3 × 10^−5^) fall into four distinct clusters: (1) the group of sociodemographic factors (including education), lifespan traits, smoking, CAD and lung cancer; (2) HDL-related traits; (3) the cluster of obesity-related traits including BMI and (4) Type 2 diabetes-related traits. The healthspan itself clusters together with CAD and parental age at death (a sub-cluster of cluster 1). We note, however, the absence of any substantial genetic correlation between the healthspan and Alzheimer disease (*r*_g_ = −0.03, Supplementary Data 8).

### Functional annotation in-silico

For the five replicated loci we selected SNPs that most likely include the functional variant (99% credible set). In total, we picked 924 SNPs (Supplementary Data 9) for further variant effect predictor analysis. The results of the variant effect predictor^31^ annotation are presented in Supplementary Data 10. We observed missense variants for some transcripts of *HLA-DQA1*, *HLA-DQB1*, *LPA*, *MC1R* (*TUBB3* exon 1), *SPATA33*, and *CASP8* genes.

DEPICT^32,33^ analysis using first the 14 “top” SNPs from Supplementary Data 5, and then a larger set of 135 independent SNPs with *p* ≤ 10^−5^ (Supplementary Data 11) did not yield any significant gene-sets or tissues/cells types enrichment, or prioritized genes (all *FDR* > 0.2, Supplementary Data 11). We have also applied DEPICT to separately analyze GWAS of the cancer-alone and non-cancer-major-diseases outcomes. Similar to the healthspan, we did not observe any significant results (all FDR > 0.2) for non-cancer-major-disease, and did not observe gene-sets enrichment or prioritized genes for cancer-alone. We did however observed tissue expression enrichment for cancer-alone; namely, “fetal blood” (hemic and immune systems) for SNP selection threshold of 5e-8 and nine tissues–with oropharynx (respiratory system) being the most significant–for SNP selection threshold of 10^−5^ at FDR < 0.2, see Supplementary Data 12 and 13.

Finally, we investigated the overlap between associations obtained here and elsewhere, using the phenoscaner v1.1 database^34^. For the 12 most significant SNPs (Table 2) we looked up traits that have demonstrated genome-wide significant (*p* < 5 × 10^−8^) associations at the same or at strongly (*r*^2^ < 0.8) linked SNPs. The results are summarized in Supplementary Data 14. For the five replicated loci we observed co-associations with a number of complex traits. The loci on chromosome 2 at 202 Mb (nearest gene *ALS2CR12*) associated with melanoma skin cancer^35^ and esophageal squamous cell carcinoma^36^. Next, loci on chromosome 6 at 0.4 Mb (*IRF4*) associated with different aspects of pigmentation, such as color of skin, eye and hair, pigmentation, tanning and freckles^37,38^, but also with non-melanoma skin cancer^38^ and the mole count in cutaneous malignant melanoma families^39^. Two loci (on chromosome 6 at 161 Mb and on chromosome 9 at 22 Mb, *LPA* and *CDKN2B-AS1*, respectively) were associated with coronary artery disease, myocardial infarction, LDL and cholesterol levels^23,40^. The remaining replicated locus on chromosome 10 at 114 Mb (*TCF7L2*) was associated with glucose levels, BMI and type 2 diabetes^41,42^.

### Effects of known lifespan-associated loci onto healthspan

We have compared whether SNPs previously reported to be associated with lifespan, (extreme) longevity^7,8,12,13,43^, and disease-free survival^11^ are also associated with healthspan in our data (Supplementary Data 15). Some SNPs we tested fall into the same region and some were discovered using the same resource (UKB). After correction for multiple testing, we find that four variants (located in or near *CDKN2B*, *ABO*, *LPA*, and *HLA-DQA1*), which have been reported to be associated with (extreme) longevity in refs. ^8,13^ were also significantly associated with the healthspan. Two of these variants reached genome-wide significance and were independently discovered as healthspan loci in this study.

## Discussion

Survival free of major disease and healthspan are related, broadly and almost interchangeably used terms that are commonly understood as the age of first chronic disease, or disability-free life-expectancy^44^. In practice, there is no widely accepted definition of healthspan^45^. Practical use of the terms “healthspan” and “disease-free survival” varies depending on the scope of a research or the availability of the relevant data. For example, Walter et al.^11^ defined the disease-free survival as the time to the first of the following adjudicated events: myocardial infarction, heart failure, stroke, dementia, hip fracture, cancer, or death. In the interest of consolidating terms, in this study, we followed a more empirical, data-driven, definition of healthspan as the age of occurrence of the first prevalent disease with a discrete clinical manifestation and following Gompertz dynamics. To do so, we used the UKB clinical information and systematically investigated the incidence of the most prevalent chronic diseases. We found that the risks of the most prevalent age-related diseases (i.e., cancer, cardiovascular disease, diabetes, dementia, COPD) grow exponentially with age at nearly the same “Gompertzian” rates. The first morbidity signifies the end of the functional or disease-free period, the healthspan, and may signal a transition into a biologically or clinically distinct and relatively short-lived state, linked with the progressive accumulation of frailty, multimorbidity, and death. The manifestly close relation between the prevalent chronic diseases and mortality suggests that the healthspan may be a very relevant aging phenotype.

Since gene variant contributions to health-span and life-span are usually small, we obtained the corresponding effect size and test statistics with the help of a simple perturbative procedure first proposed in ref. ^46^ and adopted here. It resembles a regression of the independent variable (the gene variant, in our case) against the martingale residuals of the proportional hazard model, the difference between the predicted and the observed morbidity, see, e.g., ref. ^12^. We obtained explicit analytic expressions for the regression coefficient and statistics for the specific case of parametric Cox-Gompertz mortality model, see Eqs. (2) and (3). We suggest using the proposed equations or the relevant generalizations for non-parametric risk models for fast and accurate statistical analysis involving small survival effects.

Using healthspan for quantitative studies relies on the availability of the accurate information regarding the age corresponding to onset of the diseases involved. The actual date, however, may never be known. Diagnosis always lags behind onset, and the difference may lead to a systematic bias towards later ages for diseases with gradual or hidden symptoms. MI, stroke and death from our list of morbidities have the smallest possible lag between the condition onset and corresponding diagnosis/event. Conversely, cancer, dementia, COPD, CHF and diabetes may develop gradually and hence it is difficult to obtain accurate age corresponding to the onset of these conditions. The discrepancy between the the actual and the reported ages is random and yet, for large enough cohorts, the incidence statistics should still provide a good estimation of real incidence rates. Moreover, the events are defined based on information coming from multiple sources, such as registries, hospital records, and interviews, which introduces additional sources of bias^47–49^, again, in morbidity-specific fashion. Altogether, the lack of the exact timing of the events is likely to introduce additional noise (thus somewhat decreasing the power of our analyses), while possible biases introduced by collection of disease incidence information from multiple heterogeneous sources may introduce some—most likely, negative—bias in incidence rate estimates. Also, on the technical side, the replication sample included people with different ethnicity, similar to ref. ^12^. This allowed us to achieve larger size of replication sample, hopefully, increasing the power of replication, although it can bias the results toward the confirmation of effects that are common to different human populations. At the same time, the total size of non-European ancestry sub-sample was much smaller than of the European ancestry (15,215 vs. 81,099), and we expect the bias, if any, to be small.

Since the first morbidity risk grows exponentially with age, we proposed to employ the probabilistic language of Cox-Gompertz proportional hazard model to test for associations between the demographic and genetic variables, on the one hand, and healthspan, on the other. For example, the Cox-Gompertz model estimates that the healthspan is 2.5 years lower for males than females, while the lifespan difference—using the same methods and cohort–is estimated as 3.2 years. Indeed, females in the UK (the population relevant to this study) live longer than males, although the gap between the sexes has decreased over time and is now 3.7 years^50^. The number is very close to our healthspan difference estimate. It is therefore intriguing to see if this numerical coincidence is a model artifact, or if indeed the observed difference in the lifespans could be attributed to the difference in healthspan. Four of the 12 loci identified here as associated with healthspan demonstrated significant differences of effects between males and females, see Supplementary Data 16. The observed difference could be a starting point for contemplating the significant sex-specific difference of lifespan extending effects of the same therapies typically observed in experiments in mice, see e.g., refs. ^51,52^.

It is tempting to consider the results of our GWAS as informative for potential anti-aging targets. The healthspan, as well as lifespan, however, is an integrated quantity and therefore may depend on the gene activation patterns during subsequent development stages and/or associated with life-long exposure. Therefore, our GWAS ‘hits’ may not necessarily be good targets for an intervention at advanced ages. The appearance of significant genetic correlations with such traits as the years of schooling (*p* = 5.74 × 10^−33^) and the age of the first birth (*p* = 2.37 × 10^−22^) could be indicators of such possibilities. One possible way to deconvolute the effects of human development, diseases and longevity could thus involve using longitudinal clinical data to see if there are gene variants responsible for the rate of aging or biological aging acceleration separately in every age group to negate the effects of accumulation in the course of development.

Overall, the strongest genetic correlate of the healthspan is parental longevity. More specifically, *HLA-DQB1*, *LPA*, and *CDKN2B* loci identified in relation to healthspan in this study were recently associated with parental longevity, a proxy for lifespan, in ref. ^13^. Such overall correlation and specific overlap is indeed a desired property of an aging-associated phenotype. Other traits, belonging to the same cluster, are firstly coronary artery disease, and then lung cancer, smoking behavior, age of first birth, and years of schooling (Fig. 4). The remaining large clusters correspond to traits associated with type 2 diabetes, obesity and lipid metabolism, most of which are known to relate to biological age acceleration, see e.g., ref. ^53^. The findings thus provide further evidence suggesting that healthspan and the related diseases could be controlled by common and highly conserved evolutionary mechanisms, such as nutrient sensing and insulin signaling, most robustly implicated in longevity studies in model animals^1,54^.

In order to test if the observed genetic correlation between healthspan and lifespan may be driven by the inclusion of the death events in the healthspan definition (1.7% of events), we re-run the GWAS considering death as a censoring event. The results changed only marginally. For example, the genetic correlations of newly defined healthspan with individual lifespan, parental, maternal and paternal age at death, became 0.80, −0.74, −0.65, and −0.74, respectively (which is very close to our original results of 0.82, −0.76, −0.66, and −0.74). All the twelve loci that were genome-wide significant (Table 2) were significant in this analysis as well.

The notable absence in our study of the gene variants around the *APOE* locus known for association with early onset of Alzheimers disease^55^ requires special consideration. First, as shown in Fig. 1, dementia occurs later in life and its incidence rate appears to grow faster than that of the other diseases investigated here in relation with healthspan. The estimated risk doubling time is shorter and is closer to 5 years, in agreement with, e.g., ref. ^56^. Next, we performed the dementia GWAS in the same UKB cohorts and failed to produce strong genetic correlations with the healthspan (Fig. 3; note, however, the appreciable correlation between the dementia and mortality traits). We also note the absence of significant genetic correlations between our healthspan and the non-UK Biobank-based Alzheimer GWAS^57^ (Fig. 4). These findings could be an artifact of the age composition of our discovery cohort leading to possible under-representation of dementia incidence and its influence on healthspan. It could be, however, an indication of distinct underlying biology between the late life neurodegenerative conditions and the more prevalent diseases of aging occurring at the earlier age, corresponding to the average lifespan in the population. The latter is in line with independent findings that genetic correlations between dementia and cardio-metabolic diseases is low^58^. Also, there has been reported a lack of direct effect of polygenic risk score for coronary artery disease onto dementia^59^. The absence of the associations in the *APOE* locus is potentially an important example of the differences between the genetic signatures of the healthspan and lifespan (in the form of parental survival^12^ and parental age at death^29^). While genetic correlation between these traits is high (*ρ* > 0.7), the remaining ‘uncoupled’ variance leaves room for genetic variants affecting healthspan and lifespan (parental survival) in distinct ways.

The genetic loci associated with healthspan and identified in this study together comprise the simplest form of a genetic risk model to predict early onset of chronic diseases or the age of serious disability. We used the same statistical model to perform GWAS for every ailment from our “Gompertzian” diseases list. Our analysis shows that there are at least three loci simultaneously associated with risk of multiple diseases or death and as such could be a part of the genetic signature of aging. *HLA-DQB1* is significantly (*p* = 4.18 × 10^−8^) associated with COPD, diabetes, cancer and dementia in this study and was demonstrated to be associated with parental survival earlier in ref. ^13^. The gene variant near *TYR* are predictors of death in the prospective UKB cohort and has been implicated in earlier onset of macular degeneration, a notable example of age-related disease^60^, not present in our healthspan definition. Most notably, the chromosme 20 locus containing *C20orf112* was not associated with the incidence of any of the disease at the full-genome level (see Supplementary Data 15), and yet is discovered in our healthspan GWAS.

On a population level, factors such as social status, sleep patterns or food habits produce a very significant contribution to longevity^61^, and yet are not not easy to collect and hence are hard to include in most forms of genetic studies. Modern large population studies involve prospective cohorts and produce a very rich characterization of the participants, yet at the expense of limited follow-up times and an insufficient number of recorded death events. The end of healthspan comes, by its very nature, earlier than the end of lifespan, and therefore allows for predictions to be made on the living. The healthspan as the target phenotype should thus be particularly suited for investigation of the effects of interactions between the genetic and phenotypic variables and eventually assist in the discovery of many more genes implicated in the control of human aging and diseases.

The burden of diseases increases with age, and the first morbidity is usually quickly followed by the second and more. Therefore it is worthwhile to understand if the same or different genes than those regulating the onset of the first morbidity (the end of healthspan, as defined in this study) also control the dynamics of multiple morbidities later down the road. The comparison and better understanding of the results of such studies will help to differentiate the biology of health- and life-span. Human development and aging is a multi-stage process, and therefore longevity emerges as a genuinely complex trait. The presented study highlights a need for further systematic advances in aging GWAS methodology to elucidate the practical potential of genetics in diagnosis of aging and, subsequently, help to shape the anti-aging therapeutic target space.

## Methods

### UK Biobank

UK Biobank is a prospective cohort study of over 500,000 individuals from across the United Kingdom^62^. Participants, aged between 37 and 73, were invited to one of 22 centers across the UK between 2006 and 2010. Blood, urine and saliva samples were collected, physical measurements were taken, and each individual answered an extensive questionnaire focused on questions of health and lifestyle. All participants gave written informed consent and the study was approved by the North West Multicentre Research Ethics Committee. UKB has Human Tissue Authority research tissue bank approval, meaning separate ethical approvals are not required to use the existing data. UKB provided genotyping information for 488,377 individuals. Data access to UKB was granted under application #21988. Phenotypes and genotypes were downloaded directly from UKB.

### Genotyping and imputations

UKB participants were genotyped on two slightly different arrays and quality control was performed by UKB^63^. 49,950 samples were genotyped as part of the UK BiLEVE study using a newly designed array, with 438,427 remaining samples genotyped on an updated version (UK Biobank Axiom array), both manufactured by Affymetrix (96% of SNPs overlap between the arrays). Samples were processed and genotyped in batches approx. 5000 samples each. In brief, SNPs or samples with high missingness, multi-allelic SNPs and SNPs with batchwise departures from Hardyâ€“Weinberg equilibrium were removed from the data set. After quality control, genotypes were available for 488k subjects at 805k sites. UKB provided 40 principal components (PCs) of genetic relatedness (UKB field id 22009) and a binary assessment of whether subjects were genetically confirmed European Ancestry (UKB field id 22006), based on principal components analysis of their genetic data.

We have computed Pearson correlations between self reported ethnicity (UKB field id 21000), coded as binary variable, and the 40 principal components in UKB data set of 488,363 participants with genetics principal components analysis data available. The estimates could be found in Supplementary Data 17.

Imputed data were prepared by UKB. In summary, autosomal phasing was carried out using a version of SHAPEIT3^64^ modified to allow for very large sample sizes. Imputation was carried out using IMPUTE2^65^ using the merged UK10K and 1000 Genomes Phase 3 reference panels to yield higher imputation accuracy of haplotypes. The imputations resulted in 92,693,895 SNPs, short indels and large structural variants, imputed in 488,377 individuals^63^.

### Discovery and replication samples

For the discovery and replication we used only the data from PCA cohort (QC passed, Data-Field 22020, *N* = 407,208). This cohort also represents the largest possible unrelated individuals subset^63^ with all relatives of third degree or closer removed. For the discovery set we selected 300,447 genetically confirmed white (GCW) British individuals according to the genetic principal components provided by the UK Biobank who were not included in UK BiLEVE study (UKB Resource 531). For replication, we used a combination of the UK Biobank participants not included in the discovery set that comprised rest of European ancestry individuals (self-reported white, data-field 21000, *n* = 81,099), individuals of African ancestry (self-reported Africans, *n* = 3073), individuals of South Asian ancestry (Indian, Pakistani, and Bangladeshi; *n* = 6921), Chinese individuals (*n* = 1422) and Caribbean individuals (*n* = 3799). Remaining self-declared ethnicities that were mixed, or were ambiguous (Other ethnic group, Prefer not to answer, Not available) were not analyzed. To reduce the risk of bias due to population stratification, all groups were analyzed separately followed by a meta-analysis. Total resulting sample size for replication was 96,313 individuals. Additionally, we checked that there is no individuals with kinship coefficient *r* > 0.01 between discovery and replication cohorts, using relationship data provided by UKB (UKB data category 100315). For more details see Supplementary Data 18.

The replication threshold was set as *p* < 0.05/12 = 0.004. For each SNP, statistical power (or probability) of replication was estimated using the fact that under alternative hypothesis (*H*_1_:*β* ≠ 0) the test statistics *T*^2^ from replication sample is expected to follow the $\chi _{{\mathrm{df}} = {\mathrm{1,NCP}}}^2$ distribution, where NCP is the expected non-centrality parameter computed as $(T_{{\mathrm{disc}}}^2 - 1) \times N_{{\mathrm{rep}}}/N_{{\mathrm{disc}}}$, where $T_{{\mathrm{disc}}}^2 = (\beta _{{{\mathrm{disc}}}}/se_{{\mathrm{disc}}})^2/\lambda _{{\mathrm{LDSC}}}$ is test statistic for particular SNP in discovery cohort, corrected for LD score regression interecept *λ*_LDSC_, *N*_rep_ is the sample size of the replication cohort and *N*_disc_ is the sample size of the discovery cohort. The the power of replication is equal to the probability that such distributed statistics would exceed the threshold value *k* = 8.2 that corresponds to right-hand integral of $\chi _1^2$ equal to 0.004.

### Incidence of diseases calculation from UKB data

We used in-patient hospital admissions data (UKB data category 2000) and self-reported diagnoses obtained via verbal interview (UKB data category 100074) to extract information in relation to the disease history, the nature of and the age at the available diagnosis. For each of the condition, we follow the instructions similar to the ones given by the UK Biobank outcome adjudication group for algorithmic-defined stroke and MI (UKB data category 42). For each selected condition, except for cancer and death we compile a list of hospital data codes (ICD-10, Supplementary Data 19) and self-reported data codes (UKB data coding 6) that defines these conditions in our study. We used National cancer registries linkage to UKB (UKB data category 100092) in addition to hospital data for cancer and National death registries linkage to UKB (UKB data category 100093) to define death event. First, for each condition we set the age of first occurrence of any of corresponding hospital data codes as age this condition was manifested. Next, if there was missing hospital data (for hospital data it is impossible to distinguish between missing data and absence of any disease) we added self-reported data if there was any. Therefore we obtained age each condition was occurred. The minimal age from this data set for every individual from UKB was taken as age the healthspan terminates. When calculating disease incidence rates, each participant was counted despite the existence of any other disease earlier in life, therefore some participant may have different event times for different conditions. By definition, the incidence rate of a disease is the limit *m*(*t*) = Δ*t*^−1^*N*_d_(*t*, Δ*t*)/*N*_h_(*t*) when Δ*t* is sufficiently small. Here *t* is the age, *N*_h_(*t*) is the number of people healthy at the age *t* and *N*_d_(*t*, Δ*t*) is the number of people diagnosed between the ages *t* and *t* + Δ*t* (both *N*_h_ and *N*_d_ are presumed to be large). This definition does not rely on any specific underlying model. In practice, datasets are of limited size and the interval Δ*t* cannot be made arbitrarily small, and therefore precautions should be taken to avoid possible artifacts in the calculation. To compute the incidence rate at a given age *t*, one shall consider a set of participants *Υ*(*t*, Δ*t*) defined as those who are healthy at the age *t* and whose health status is available in the whole age range [*t*, *t* + Δ*t*): $\Upsilon (t,{\mathrm{\Delta }}t) = \{ u|((\delta ^u = 0) \vee (\delta ^u = 1 \wedge t \le t_{\mathrm{d}}^u)) \wedge (t + {\mathrm{\Delta }}t \ < \ t_2^u)\}$. Here *u* is the participant’s id, *δ*^*u*^ = 1 if the participant was diagnosed and *δ*^*u*^ = 0 otherwise, $t_{\mathrm{d}}^u$ is the age when diagnosed, and $t_2^u$ is the maximal age at which the information about the diagnosis (if any) would still be recorded. From this *N*_h_(*t*) = |*Υ*(*t*, Δ*t*)| and $N_{\mathrm{d}}(t,{\mathrm{\Delta }}t) = |\{ u \in \Upsilon (t,{\mathrm{\Delta }}t)|\delta ^u = 1 \wedge t \le t_{\mathrm{d}}^u \ < \ t + {\mathrm{\Delta }}t\} |$, where |..| is the size of the set.

The maximum follow-up age $t_2^u$ does not coincide with the age at the diagnosis $t_{\mathrm{d}}^u$ and shall be inferred from the study setup. Assuming $t_2^u = t_{\mathrm{d}}^u$ for diagnosed participants would overestimate the risks. Also, the age is often rounded and hence Δ*t* may be not large enough to treat the rounding errors as negligible. We addressed the issue by consistently using half-open intervals [..) definitions. Finally, our prescription relies on the implicit assumption, that the diagnosis does not influence the enrollment. This is not always true. If someone is dead, this would, naturally, prevent that person from being enrolled at a greater age. This can be addressed by the following modification: $\Upsilon \prime (t,{\mathrm{\Delta }}t) = \{ u\, \in \,\Upsilon (t,{\mathrm{\Delta }}t)|t_1^u \ < \ t\}$, where $t_1^u$ is the age at enrollment. In this study, we assumed that the enrollment in UKB was not biased by diagnoses and thus we used the *Υ* for all diseases and conditions, *Υ*^'^ participants set was only employed for the mortality rate calculation.

### Cox-Gompertz proportional hazards model and healthspan

By design of the UKB study, every participant is admitted into the cohort at the age $t_1^n$. According to the medical history information, the participant may be diagnosed with any of the diseases relevant to determination of lifespan at the age of the first $t_{\mathrm{d}}^n$ (if applicable). By the end of the followup age, $t_2^n$, we labeled every study participant as frail, *δ*^*n*^ = 1, if the participant is already diagnosed with any of the diseases, $t_{\mathrm{d}}^n \le t_2^n$, or *δ*^*n*^ = 0, otherwise.

Under then Cox-Gompertz proportional hazards model the risks of frailty acquisition or healthspan end at the age *t* is $h(t,x^n) = h_0e^{{\mathrm{\Gamma }}t + \beta x^n}$, where *x*^*n*^ is a vector of age-independent parameters, characterizing the participant. Here *h*_0_, *Γ*, and *β* are the baseline morbidity incidence, the Gompertz exponent and the log-odds-ratio regression coefficients vector, the model parameters. The (negative log of) likelihood of the data can be presented in the following form:

1 $$\begin{array}{*{20}{l}} L \hfill & = \hfill & {\mathop {\sum}\limits_n \frac{{h_0e^{\beta x^n}}}{\Gamma }\left( {e^{{\mathrm{\Gamma }}\,{\mathrm{min}}\left( {t_{\mathrm{d}}^n,t_2^n} \right)} - 1} \right)} \hfill \\ {} \hfill & {} \hfill & { - \delta ^n({\mathrm{log}}\,h_0 + \beta x^n + {\mathrm{\Gamma }}\,{\mathrm{min}}(t_{\mathrm{d}}^n,t_2^n)).} \hfill \end{array}$$

Given a necessary amount of data the model parameters could be obtained by the likelihood maximization or, equivalently, minimization of the cost function *L*.

We built the first version of the Cox-Gompertz healthspan model by including GCW-British UKB participants information, including gender and the first genetic principal components variables, assessment center codes and genotyping batch labels (see Supplementary Data 3 for the summary of the model parameters). The morbidity incidence growth rate is 0.098 per year, which corresponds to a doubling time of seven years, compatible with the mortality rate doubling time of approximately 8 from the Gompertz mortality law. As expected, being male is a risk factor (log-hazard ratio, log(HR) = 0.26 at the significance level of *p* = 5 × 10^−301^) corresponding to an average healthspan difference of about five years. The genetic principal component PC4 was highly significant log(HR) = 3.4 × 10^−2^, *p* = 9.2 × 10^−23^. PC5 was also highly significant log(HR) = 4.6 × 10^−2^, *p* = 1.7 × 10^−40^. The average healthspan or lifespan can be estimated from Cox-Gompertz model parameters as $\bar t \approx ({\mathrm{ln}}({\mathrm{\Gamma }}/h_0) - \gamma )/{\mathrm{\Gamma }}$, where *γ* = 0.577 is the Euler-Mascheroni constant, see, e.g.,^66^.

### Gene variant-healthspan association testing

If the participants state vector $x_i^n$ is extended by the genetic variants variables *s*^*n*^, in principle, the model has to be re-evaluated, to obtain a new versions of all model parameters. We do not expect, however, large effects of any of the gene variants on lifespan. Therefore the model parameters should not change much as well and the variation of the Cox-Gompertz model with respect to the genetic variables can be accurately obtained by iterations, using the model from 4.5 as the zeroth order approximation (see a related example of a perturbation theory application in a proportional hazards model involving prediction of all-cause mortality in ref. ^46^).

We note, however, that the simultaneous determination of the weak effects of a gene on the baseline hazard *h*_0_ and the rate of aging *Γ* is an ill-defined mathematical problem^66^. Only the combination of the two parameters, the change in the life expectancy can be determined with accuracy. We therefore fix the Gompertz exponent Γ to its most probable value in the zeroth order model and allow for all other model parameters adjustment. The perturbation theory expansion for the small effect *β*_*s*_ associated with the gene variants yields (the derivation is not shown):

2 $$\beta _s = \frac{{\mathop {\sum}\nolimits_n {s^n} \left( {\delta ^n - N_{\mathrm{d}}\rho ^n} \right)}}{{N_{\mathrm{d}}\left\langle {\delta s^2} \right\rangle _\rho }},$$

where, for convenience, we introduced the weights

$$\rho ^n = \frac{{e^{\beta x^n}\left( {e^{{\mathrm{\Gamma }}\,{\mathrm{min}}\left( {t_{\mathrm{d}}^n,t_2^n} \right)} - 1} \right)}}{{\mathop {\sum}\limits_n e^{\beta x^n}\left( {e^{{\mathrm{\Gamma }}\,{\mathrm{min}}\left( {t_{\mathrm{d}}^n,t_2^n} \right)} - 1} \right)}}$$

normalized in such a way that $\mathop {\sum}\nolimits_n \rho _n = 1$. We used the notation 〈*δs*^2^〉_*ρ*_ for the corresponding weighted average. The effect determination error

3 $$\sigma _s^2 = \frac{1}{{N_{\mathrm{d}}\left\langle {\delta s^2} \right\rangle _\rho }},$$

and hence the statistical power of the gene variant association with the healthspan is explicitly dependent on the number of people with diagnoses, $N_{\mathrm{d}} = \mathop {\sum}\nolimits_n \delta ^n$.

In our analyses, we used imputed variants with the expected effective minor allele count (defined as twice the minor allele frequency multiplied by sample size and by the imputation quality) more than 200 for discovery cohort genotypes and imputation info score (as IMPUTE info, calculated by RegScan^67^ for discovery cohort with–info2 option) more than 0.7.

### Conditional and joint multi-SNP analysis

Conditional and joint analysis (COJO) as implemented in the program GCTA^21^ was used to find SNPs independently associated with the phenotypes of interest. As input, this method uses (meta-analysis) summary statistics and a reference sample that is utilized for the LD estimation. The method starts with the “top SNP” (the one with smallest p-value, conditional that *p* < *p*_0_, where *p*_0_ is specific threshold defined by user) as provided by the summary-level data and then the p-values for all the remaining SNPs are calculated conditional on the selected SNP. The algorithm then selects the next top SNP in the conditional analysis (provided *p* < *p*_0_) and proceeds to fit all the selected SNPs in the model dropping all those SNPs with p-values > *p*_0_. The iteration continues until no SNP is added or dropped from the model thus finding a subset of associated SNPs with a threshold for LD (*r*^2^ < 0.9) among SNPs. Finally, a joint analysis of the subset of associated SNPs is performed. We had performed analyses with *p*_0_ = 5 × 10^−8^ and *p*_0_ = 1 × 10^−5^.

As the LD reference, we used a sub-sample of 10,000 people, randomly chosen from the total set of 120,286 people used for GWAS discovery phase. Additional to our previous SNP filters described in the Association testing section, in selecting LD reference data, we further filtered out the SNPs with imputation info scores less than 0.7 and minor allele frequencies (MAF) less than 0.002.

### Sex-specific analysis

We performed sex-specific genetic association analysis (males: *n* = 137,469, females: *n* = 162,978) for 12 genome-wide significantly associated SNPs from Table 2. We estimated the difference of SNP effects between males and females using approach from ref. ^68^ (see “SNP selection strategy” subsection in Methods, Eq. (1)) that allows testing difference between effect sizes accounting for their possibly correlated joint distribution. The results are reported in Supplementary Data 16. For this method Spearman correlation for effect sizes between males and females was estimated using only called SNPs with MAF > 0.05 (377,781 SNPs in total). The significance threshold was set as *p* < 0.05/12 = 0.042.

### Heritability and genetic correlation analyses

We used LD hub and ldsc^58^ tools for estimation of captured heritability and genetic correlations between HS and different traits and common diseases^58^. A total of 231 traits were analyzed after removing duplicates via using only the most recent study for each trait as indicated by the largest PMID number. Genetic correlations between HS and the traits with *p* < 4.3 × 10^−5^ (Bonferroni corrected, 0.01/231) were considered statistically significant. Pair-wise genetic correlations between all the traits selected as described above were obtained from the LD-hub. To focus on the largest magnitude genetic correlations, we selected only the traits with absolute values of genetic correlations with HS more than 0.3. This filtering led to the total of 36 traits (including HS). Clustering and visualization was carried out using corrplot package for R and basic hclust function. For clustering, we estimated squared Euclidean distances by subtracting absolute values of genetic correlation from 1 and used Ward’s clustering method.

For genetic correlation analysis between each disease comprising healthspan phenotype and healthspan itself we used LDSC (LD Score) v1.0.0 software. Genotype calls were filtered by MAF > 0.01 using LDSC ‘munge-sumstats’ script to produce total 659,079 variants valid for downstream analysis. Genomic reference was constructing by randomly sampling 10,000 individuals from the UKB population. Then, we ran LDSC genetics correlation analysis with default parameters and input data as described above. Cross-correlations can be seen at Fig. 3 and Supplementary Data 16.

For analysis of heritability, genomic control inflation factor *λ*^19^ and genetics correlations we have used SNPs defined by overlap between our set of SNPs and ‘high quality SNPs’ as suggested by the authors of the LD hub (these represent common HapMap3 SNPs that usually have high imputation quality; also, this set excludes HLA region)^20^, 1,162,742 SNPs in total).

### Variant effect prediction (VEP)

We used PAINTOR software^69^ to prepare the set of SNPs for VEP annotation. For this analysis, we provided PAINTOR with clumping results, LD matrices and annotation files calculated using the same 10,000 UKB individuals reference set that we used for COJO analysis. With PLINK^70^ and we performed clumping analysis with ‘*p*1’ and ‘*p*2’ *p*-value threshold parameters set to 5 × 10^−8^, ‘*r*2’ set to 0.1 and *MAF* > 0.002. Then, we generated pair-wise correlation matrix for all SNPs in each region in clumping analysis results using plink–r option. When running PAINTOR, we did not use annotations; we changed options controlling input and output files format only, and otherwise we have used default parameters. We choose 159 SNPs marked by PAINTOR as 99% credible set for further analysis. In the next step, each SNP was extended with a list of proxy SNPs with *R*2 > 0.8 calculated using EUR cohort from 1000 Genomes Project Phase 3^71^ (*N* = 503) with 84.4 million variants as reference set. Total 924 SNPs was chosen for functional annotation by VEP with GRCH37 genomic reference.

### Gene-set and tissue/cell enrichment analysis

For prioritizing genes in associated regions, gene set enrichment and tissue/cell type enrichment analyses, we have used the DEPICT software v. 1 rel. 194^32^ with following parameters: flag_loci = 1; flag_genes = 1; flag_genesets = 1; flag_tissues = 1; param_ncores = 10. Independent (as selected by COJO procedure) variants with *p* < 5 × 10^−8^ (14 SNPs) and *p* < 10^−5^ (135 SNPs) has resulted from these analyses. We have used UKB subset of 10,000 individuals for computations of LD (the same subset as used for COJO analysis).

### Pleiotropy with complex traits

We investigated the overlap between associations obtained here and elsewhere, using PhenoScaner v1.1 database^34^. For five replicated SNPs (Table 1) we looked up traits that have demonstrated genome-wide significant (*p* < 5 × 10^−8^) association at the same or at strongly (*r*2 < 0.8) linked SNPs.

### Code availability

All computer code used in this research is available at https://github.com/azenin/healthspanpaper.

### Reporting Summary

Further information on experimental design is available in the Nature Research Reporting Summary linked to this Article.

## Supplementary Information

Reporting Summary

Description of Additional Supplementary Files

Supplementary Data 1

Supplementary Data 2

Supplementary Data 3

Supplementary Data 4

Supplementary Data 5

Supplementary Data 6

Supplementary Data 7

Supplementary Data 8

Supplementary Data 9

Supplementary Data 10

Supplementary Data 11

Supplementary Data 12

Supplementary Data 13

Supplementary Data 14

Supplementary Data 15

Supplementary Data 16

Supplementary Data 17

Supplementary Data 18

Supplementary Data 19

## Data Availability

All UK Biobank data are available upon application. Summary statistics from the GWAS reported in this study are available for exploration at GWASarchive (https://www.gwasarchive.org) and for download from Zenodo^72^ under the CC BY 4.0 license.

## References

[1] Niccoli T, Partridge L (2012). Ageing as a risk factor for disease. Curr. Biol..

[2] Andersen SL, Sebastiani P, Dworkis DA, Feldman L, Perls TT (2012). Health span approximates life span among many supercentenarians: compression of morbidity at the approximate limit of life span. J. Gerontol. Ser. A.

[3] Kennedy BK (2014). Aging: a common driver of chronic diseases and a target for novel interventions. Cell.

[4] Partridge L, Deelen J, Slagboom PE (2018). Facing up to the global challenges of ageing. Nature.

[5] Vijg J, Suh Y (2005). Genetics of longevity and aging. Annu. Rev. Med..

[6] Jagger C (2008). Inequalities in healthy life years in the 25 countries of the european union in 2005: a cross-national meta-regression analysis. Lancet.

[7] Deelen J (2014). Genome-wide association meta-analysis of human longevity identifies a novel locus conferring survival beyond 90 years of age. Human. Mol. Genet..

[8] Fortney K (2015). Genome-wide scan informed by age-related disease identifies loci for exceptional human longevity. PLoS Genet.

[9] Zeng Y (2016). Novel loci and pathways significantly associated with longevity. Sci. Rep..

[10] Slagboom, P. E., van den Berg, N. & Deelen, J. Phenome and genome based studies into human ageing and longevity: an overview. *Biochim. Biophys. Acta* **1864**, 2742–2751 (2018). 10.1016/j.bbadis.2017.09.017 28951210

[11] Walter S (2011). A genome-wide association study of aging. Neurobiol. Aging.

[12] Joshi PK (2016). Variants near chrna3/5 and apoe have age-and sex-related effects on human lifespan. Nat. Commun..

[13] Joshi PK (2017). Genome-wide meta-analysis associates hla-dqa1/drb1 and lpa and lifestyle factors with human longevity. Nat. Commun..

[14] McDaid AF (2017). Bayesian association scan reveals loci associated with human lifespan and linked biomarkers. Nat. Commun..

[15] Gompertz B (1825). On the nature of the function expressive of the law of human mortality, and on a new mode of determining the value of life contingencies. Philos. Trans. R. Soc. Lond..

[16] Makeham WM (1860). On the law of mortality and construction of annuity tables. Assur. Mag. J. Inst. Actuar..

[17] Schemper M, Kaider A, Wakounig S, Heinze G (2013). Estimating the correlation of bivariate failure times under censoring. Stat. Med..

[18] Tutkun NA, Demirhan H (2016). A bayesian approach to cox-gompertz model. Hacet. J. Math. Stat..

[19] Devlin B, Roeder K (1999). Genomic control for association studies. Biometrics.

[20] Bulik-Sullivan BK (2015). Ld score regression distinguishes confounding from polygenicity in genome-wide association studies. Nat. Genet..

[21] Yang J (2012). Conditional and joint multiple-snp analysis of gwas summary statistics identifies additional variants influencing complex traits. Nat. Genet..

[22] Zheng J (2017). Ld hub: a centralized database and web interface to perform ld score regression that maximizes the potential of summary level gwas data for snp heritability and genetic correlation analysis. Bioinformatics.

[23] Nikpay M (2015). A comprehensive 1000 genomes–based genome-wide association meta-analysis of coronary artery disease. Nat. Genet..

[24] Morris AP (2012). Large-scale association analysis provides insights into the genetic architecture and pathophysiology of type 2 diabetes. Nat. Genet..

[25] Soranzo N (2010). Common variants at 10 genomic loci influence hemoglobin a1c levels via glycemic and nonglycemic pathways. Diabetes.

[26] Furberg H (2010). Genome-wide meta-analyses identify multiple loci associated with smoking behavior. Nat. Genet..

[27] Dupuis J (2010). New genetic loci implicated in fasting glucose homeostasis and their impact on type 2 diabetes risk. Nat. Genet..

[28] Barban N (2016). Genome-wide analysis identifies 12 loci influencing human reproductive behavior. Nat. Genet..

[29] Pilling LC (2016). Human longevity is influenced by many genetic variants: evidence from 75,000 uk biobank participants. Aging.

[30] Kettunen J (2016). Genome-wide study for circulating metabolites identifies 62 loci and reveals novel systemic effects of lpa. Nat. Commun..

[31] McLaren W (2016). The ensembl variant effect predictor. Genome Biol..

[32] Pers TH (2015). Biological interpretation of genome-wide association studies using predicted gene functions. Nat. Commun..

[33] Consortium GP (2010). A map of human genome variation from population-scale sequencing. Nature.

[34] Staley JR (2016). Phenoscanner: a database of human genotype–phenotype associations. Bioinformatics.

[35] Barrett JH (2011). Genome-wide association study identifies three new melanoma susceptibility loci. Nat. Genet..

[36] Abnet CC (2012). Genotypic variants at 2q33 and risk of esophageal squamous cell carcinoma in china: a meta-analysis of genome-wide association studies. Human. Mol. Genet..

[37] Eriksson N (2010). Web-based, participant-driven studies yield novel genetic associations for common traits. PLoS Genet..

[38] Zhang M (2013). Genome-wide association studies identify several new loci associated with pigmentation traits and skin cancer risk in european americans. Human. Mol. Genet..

[39] Duffy DL (2010). Irf4 variants have age-specific effects on nevus count and predispose to melanoma. Am. J. Human. Genet..

[40] Willer CJ (2013). Discovery and refinement of loci associated with lipid levels. Nat. Genet..

[41] Gaulton KJ (2015). Genetic fine mapping and genomic annotation defines causal mechanisms at type 2 diabetes susceptibility loci. Nat. Genet..

[42] Shungin D (2015). New genetic loci link adipose and insulin biology to body fat distribution. Nature.

[43] Broer L (2014). Gwas of longevity in charge consortium confirms apoe and foxo3 candidacy. J. Gerontol. Ser. A.

[44] Crimmins EM (2015). Lifespan and healthspan: past, present, and promise. Gerontologist.

[45] Kaeberlein, M. How healthy is the healthspan concept? *GeroScience* **40**, 1–4 (2018). 10.1007/s11357-018-0036-9 PMC6136295 30084059

[46] Pyrkov TV (2018). Extracting biological age from biomedical data via deep learning: too much of a good thing?. Sci. Rep..

[47] Papoz L, Balkau B, Lellouch J (1996). Case counting in epidemiology: limitations of methods based on multiple data sources. Int. J. Epidemiol..

[48] Yoshihara H, Yoneoka D (2014). Understanding the statistics and limitations of large database analyses. Spine.

[49] Izquierdo JN, Schoenbach VJ (2000). The potential and limitations of data from population-based state cancer registries. Am. J. Public Health.

[50] Sanders, S. National life tables, UK: 2014 to 2016. https://www.ons.gov.uk/peoplepopulationandcommunity/birthsdeathsandmarriages/lifeexpectancies/bulletins/nationallifetablesunitedkingdom/2014to2016, *ONS* (2017).

[51] Harrison DE (2014). Acarbose, 17-*α*-estradiol, and nordihydroguaiaretic acid extend mouse lifespan preferentially in males. Aging Cell.

[52] Strong R (2016). Longer lifespan in male mice treated with a weakly estrogenic agonist, an antioxidant, an *α*-glucosidase inhibitor or a nrf2-inducer. Aging Cell.

[53] Horvath S (2014). Obesity accelerates epigenetic aging of human liver. Proc. Natl Acad. Sci..

[54] Piper M, Selman C, McElwee J, Partridge L (2008). Separating cause from effect: how does insulin/igf signalling control lifespan in worms, flies and mice?. J. Intern. Med..

[55] Roses A (2010). A tomm40 variable-length polymorphism predicts the age of late-onset alzheimer’s disease. Pharm. J..

[56] Ziegler-Graham K, Brookmeyer R, Johnson E, Arrighi HM (2008). Worldwide variation in the doubling time of alzheimer’s disease incidence rates. Alzheimer’s Dement..

[57] Lambert JC (2013). Meta-analysis of 74,046 individuals identifies 11 new susceptibility loci for alzheimer’s disease. Nat. Genet..

[58] Bulik-Sullivan B (2015). An atlas of genetic correlations across human diseases and traits. Nat. Genet..

[59] Karlsson I (2017). Genetic susceptibility to cardiovascular disease and risk of dementia. Transl. Psychiatry.

[60] Holliday EG (2013). Insights into the genetic architecture of early stage age-related macular degeneration: a genome-wide association study meta-analysis. PloS One.

[61] Wilhelmsen L (2011). Factors associated with reaching 90 years of age: a study of men born in 1913 in gothenburg, sweden. J. Intern. Med..

[62] Sudlow C (2015). Uk biobank: an open access resource for identifying the causes of a wide range of complex diseases of middle and old age. PLoS Med..

[63] Bycroft C (2018). The uk biobank resource with deep phenotyping and genomic data. Nature.

[64] O’Connell J (2016). Haplotype estimation for biobank-scale data sets. Nat. Genet..

[65] Howie B, Marchini J, Stephens M (2011). Genotype imputation with thousands of genomes. G3: Genes, Genomes, Genet..

[66] Tarkhov AE, Menshikov LI, Fedichev PO (2017). Strehler-mildvan correlation is a degenerate manifold of gompertz fit. J. Theor. Biol..

[67] Haller T, Kals M, Esko T, Mägi R, Fischer K (2015). Regscan: a gwas tool for quick estimation of allele effects on continuous traits and their combinations. Brief. Bioinforma..

[68] Randall JC (2013). Sex-stratified genome-wide association studies including 270,000 individuals show sexual dimorphism in genetic loci for anthropometric traits. PLoS Genet..

[69] Kichaev, G. et al. Improved methods for multi-trait fine mapping of pleiotropic risk loci. *Bioinformatics* **33**, 248–255 (2017). 10.1093/bioinformatics/btw615 PMC5254076 27663501

[70] Chang CC (2015). Second-generation plink: rising to the challenge of larger and richer datasets. Gigascience.

[71] Consortium GP (2015). A global reference for human genetic variation. Nature.

[72] Zenin, A. et al. Genome-wide association summary statistics for human healthspan. *Zenodo*, 10.5281/zenodo.1302861 (2018).

