# Supplementary material for: Identification of 12 genetic loci associated with human healthspan
Source: Commun Biol. 2019 Jan 30;2:41. doi: 10.1038/s42003-019-0290-0 (PMC6353874; doi:10.1038/s42003-019-0290-0)
Supplement: Supplementary file 1 — Reporting Summary [file 42003_2019_290_MOESM1_ESM.pdf]

nature research

Corresponding author(s): Peter Fedichev

Reporting Summary

Nature Research wishes to improve the reproducibility of the work that we publish. This form provides structure for consistency and transparency in reporting. For further information on Nature Research policies, see Authors & Referees and the Editorial Policy Checklist.

Statistical parameters

When statistical analyses are reported, confirm that the following items are present in the relevant location (e.g. figure legend, table legend, main text, or Methods section).

n/a	Confirmed	
☐	☒	The exact sample size (*n*) for each experimental group/condition, given as a discrete number and unit of measurement
☐	☒	An indication of whether measurements were taken from distinct samples or whether the same sample was measured repeatedly
☐	☒	The statistical test(s) used AND whether they are one- or two-sided<br>*Only common tests should be described solely by name; describe more complex techniques in the Methods section.*
☐	☒	A description of all covariates tested
☐	☒	A description of any assumptions or corrections, such as tests of normality and adjustment for multiple comparisons
☐	☒	A full description of the statistics including central tendency (e.g. means) or other basic estimates (e.g. regression coefficient) AND variation (e.g. standard deviation) or associated estimates of uncertainty (e.g. confidence intervals)
☐	☒	For null hypothesis testing, the test statistic (e.g. *F*, *t*, *r*) with confidence intervals, effect sizes, degrees of freedom and *P* value noted<br>*Give P values as exact values whenever suitable.*
☒	☐	For Bayesian analysis, information on the choice of priors and Markov chain Monte Carlo settings
☒	☐	For hierarchical and complex designs, identification of the appropriate level for tests and full reporting of outcomes
☐	☒	Estimates of effect sizes (e.g. Cohen's *d*, Pearson's *r*), indicating how they were calculated
☐	☒	Clearly defined error bars<br>*State explicitly what error bars represent (e.g. SD, SE, CI)*

Our web collection on statistics for biologists may be useful.

Software and code

Policy information about availability of computer code

Data collection	ascp version 3.7.4.147133, aws-cli/1.11.13 Python/3.5.2 Linux/4.4.0-1070-aws botocore/1.4.70, Ega Demo Download Client Version: 2.2.2, and ukbconv, ukbunpack, ukbfetch, ukbgene by UK Biobank
Data analysis	Python 2.7.12, GCTA64 version 1.91.1 beta, PAINTOR v3.0, PLINK v1.90b5.2 64-bit (9 Jan 2018), PLINK v2.00a1LM AVX2 Intel (5 Feb 2018). Custom python code available at https://github.com/azenin/healthspanpaper.

For manuscripts utilizing custom algorithms or software that are central to the research but not yet described in published literature, software must be made available to editors/reviewers upon request. We strongly encourage code deposition in a community repository (e.g. GitHub). See the Nature Research guidelines for submitting code & software for further information.

Data

Policy information about availability of data

All manuscripts must include a data availability statement. This statement should provide the following information, where applicable:

- Accession codes, unique identifiers, or web links for publicly available datasets
- A list of figures that have associated raw data
- A description of any restrictions on data availability

Summary statistics from the GWAS reported in this study are available for download from the GWAS Archive (http://gwasarchive.org) under the CC BY 4.0 license. Individual level data can be requested from UK Biobank.

Field-specific reporting

Please select the best fit for your research. If you are not sure, read the appropriate sections before making your selection.

☒ Life sciences ☐ Behavioural & social sciences ☐ Ecological, evolutionary & environmental sciences

For a reference copy of the document with all sections, see nature.com/authors/policies/ReportingSummary-flat.pdf

Life sciences study design

All studies must disclose on these points even when the disclosure is negative.

Sample size	For the discovery and replication we used only the data from PCA cohort of the UK Biobank (QC passed, Data-Field 22020, N = 407,208). For the discovery set we selected 300,447 British individuals of European ancestry (EA) according to the genetic principal components provided by the UK Biobank who were not included in UK BiLEVE study (UKB Resource 531).
Data exclusions	We do not define any exclusion criteria except these defined by UK Biobank.
Replication	For replication, we used a combination of the UK Biobank participants not included in the discovery set that comprised rest of EA individuals (self-reported white, data-field 21000, n = 81,099), individuals of African ancestry (self-reported Africans, n = 3,073), individuals of South Asian ancestry (Indian, Pakistani, and Bangladeshi; n = 6,921), Chinese individuals (n = 1,422) and Caribbean individuals (n=3,799). Remaining self-declared ethnicities that were mixed, or were ambiguous (Other ethnic group, Prefer not to answer, Not available) were not analysed. To reduce the risk of bias due to population stratification, all groups were analyzed separately followed by a meta-analysis. Total resulting sample size for replication was 96,313 individuals. The replication was considered successful.
Randomization	Randomization is not relevant to our study design.
Blinding	Blinding is not relevant to our study design.

Reporting for specific materials, systems and methods

Materials & experimental systems

n/a	Involved in the study	
☒	☐	Unique biological materials
☒	☐	Antibodies
☒	☐	Eukaryotic cell lines
☒	☐	Palaeontology
☒	☐	Animals and other organisms
☐	☒	Human research participants

Methods

n/a	Involved in the study	
☒	☐	ChIP-seq
☒	☐	Flow cytometry
☒	☐	MRI-based neuroimaging

Human research participants

Policy information about studies involving human research participants

Population characteristics	UK Biobank is a prospective cohort study of over 500,000 individuals from across the United Kingdom http://www.ukbiobank.ac.uk

Recruitment

Participants, aged between 40 and 69, were invited to one of 22 centres across the UK between 2006 and 2010 http://www.ukbiobank.ac.uk
