# Supplementary material for: Identification of 12 genetic loci associated with human healthspan
Source: Commun Biol. 2019 Jan 30;2:41. doi: 10.1038/s42003-019-0290-0 (PMC6353874; doi:10.1038/s42003-019-0290-0)
Supplement: Supplementary file 2 — Description of Additional Supplementary Files [file 42003_2019_290_MOESM2_ESM.docx]

Description of additional supplementary items

File Supplementary Data 1.xlsx

Sheet "Sheet1": Disease incidence statistics.

File Supplementary Data 2.xlsx

Sheet "cancer": Cancer incidence rate statistics.

Sheet "death": Death incidence rate statistics.

Sheet "CHF": Congestive Heart Failure incidence rate statistics.

Sheet "COPD": Chronic Obstructive Pulmonary Disease incidence rate statistics.

Sheet "MI": Myocardial Infarction incidence rate statistics.

Sheet "healthspan": Healthspan incidence rate statistics.

Sheet "stroke": Stroke incidence rate statistics.

Sheet "dementia": Dementia incidence rate statistics.

Sheet "diabetes": Diabetes incidence rate statistics.

File Supplementary Data 3.xlsx

Sheet "Sheet1": Cox-Gompertz model parameters estimation.

File Supplementary Data 4.xlsx

Sheet "Sheet1": All significant SNPs.

File Supplementary Data 5.xlsx

Sheet "Sheet1": Extended results for variants, tagging regions, significantly associated with healthspan in the discovery sample of 300,447 individuals; association between these variants and lifespan in the replication sample of 55,276 self-reported British individuals and 41,037 individuals of other ethnicities (total N=96,313).

File Supplementary Data 6.xlsx

Sheet "p": Genetic correlation p-values between healthspan and different traits, as estimated by LD score regression.

Sheet "rg": Genetic correlation coefficients between healthspan and different traits, as estimated by LD score regression.

File Supplementary Data 7.xlsx

Sheet "Sheet1": Cox-Gomperz summary statistics for the end-points that define healthspan, and for parental lifespan.

File Supplementary Data 8.xlsx

Sheet "20171208_mapping_235_rgLDHUB_GC": LDHub traits meta-data.

Sheet "20181213_gen_corrs_35x35": 35 LDHub traits genetics correlations.

Sheet "andersen_list4ldhub": Genetic correlations between healthspan and different traits, as estimated by LD score regression.

Sheet "LD_hub_rg_214x214": Genetic correlations for 214 traits from LDHub

File Supplementary Data 9.xlsx

Sheet "PAINTOR Credible Set": 99% credible set for SNPs implicated as genome-wide significant and independent by PAINTOR analysis.

Sheet "1000Genomes SNPs": 1000Genomes SNPs with R2>0.8 for PAINTOR credible set.

Sheet "SNPs for VEP analysis": Resulting list of SNPs for VEP analysis.

File Supplementary Data 10.xlsx

Sheet "Sheet1": Summary from analysis of variants from 99% credible set with variant effect predictor (VEP).

File Supplementary Data 11.xlsx

Sheet "5e-8_genesetenrichment": Gene set enrichment analysis with DEPICT on healthspan phenotype (input SNPs with p<5e-8).

Sheet "5e-8_geneprioritization": Gene prioritization with DEPICT on healthspan phenotype (input SNPs with p<5e-8).

Sheet "5e-8_tissueenrichment": Tissue enrichment analysis with DEPICT on healthspan phenotype (input SNPs with p<5e-8).

Sheet "1e-5_genesetenrichment": Gene set enrichment analysis with DEPICT on healthspan phenotype (input SNPs with p<1e-5).

Sheet "1e-5_geneprioritization": Gene prioritisation with DEPICT on healthspan phenotype (input SNPs with p<1e-5).

sheet "1e-5_tissueenrichment": Tissue enrichment analysis with DEPICT on healthspan phenotype (input SNPs with p<1e-5).

File Supplementary Data 12.xlsx

Sheet "5e-8_genesetenrichment": Gene set enrichment analysis with DEPICT on cancer-alone phenotype (input SNPs with p<5e-8).

Sheet "5e-8_geneprioritization": Gene prioritization with DEPICT on cancer-alone phenotype (input SNPs with p<5e-8).

Sheet "5e-8_tissueenrichment": Tissue enrichment analysis with DEPICT on cancer-alone phenotype (input SNPs with p<5e-8).

Sheet "1e-5_genesetenrichment": Gene set enrichment analysis with DEPICT on cancer-alone phenotype (input SNPs with p<1e-5).

Sheet "1e-5_geneprioritization": Gene prioritisation with DEPICT on cancer-alone phenotype (input SNPs with p<1e-5).

sheet "1e-5_tissueenrichment": Tissue enrichment analysis with DEPICT on cancer-alone phenotype (input SNPs with p<1e-5).

File Supplementary Data 13.xlsx

Sheet "5e-8_genesetenrichment": Gene set enrichment analysis with DEPICT on non-cancer-major-diseases phenotypeon non-cancer-major-diseases phenotype (input SNPs with p<5e-8).

Sheet "5e-8_geneprioritization": Gene prioritization with DEPICT on non-cancer-major-diseases phenotype (input SNPs with p<5e-8).

Sheet "5e-8_tissueenrichment": Tissue enrichment analysis with DEPICT on non-cancer-major-diseases phenotype (input SNPs with p<5e-8).

Sheet "1e-5_genesetenrichment": Gene set enrichment analysis with DEPICT on non-cancer-major-diseases phenotype (input SNPs with p<1e-5).

Sheet "1e-5_geneprioritization": Gene prioritisation with DEPICT on non-cancer-major-diseases phenotype (input SNPs with p<1e-5).

Sheet "1e-5_tissueenrichment": Tissue enrichment analysis with DEPICT on non-cancer-major-diseases phenotype (input SNPs with p<1e-5).

File Supplementary Data 14.xlsx

Sheet "A": Other traits associated to the regions showing significant association with healthspan (GWAS).

Sheet "B": Other traits associated to the regions showing significant association with healthspan (eQTL).

Sheet "C": Other traits associated to the regions showing significant association with healthspan (Metabolites).

File Supplementary Data 15.xlsx

Sheet "Sheet1": Variants, previously implicated in studies of longevity, aging, and disease-free survival, and their association with healthspan, ''Gomepertzian'' disease, and parental lifespan in 300,447 individuals.

File Supplementary Data 16.xlsx

Sheet "Sheet1": Sex-specific analysis of top hits.

File Supplementary Data 17.xlsx

Sheet "Sheet1": Genetic principal components correlation with self-reported ethnicity.

File Supplementary Data 18.xlsx

Sheet "Sheet1": Replication cohort composition.

File Supplementary Data 19.xlsx

Sheet "Sheet1": Disease codes for healthspan composition.
